# *Boechera* Species Exhibit Species-Specific Responses to Combined Heat and High Light Stress

**DOI:** 10.1371/journal.pone.0129041

**Published:** 2015-06-01

**Authors:** Genna Gallas, Elizabeth R. Waters

**Affiliations:** Dept. of Biology San Diego State University, San Diego, California, United States of America; Institute of Genetics and Developmental Biology, Chinese Academy of Sciences, CHINA

## Abstract

As sessile organisms, plants must be able to complete their life cycle in place and therefore tolerance to abiotic stress has had a major role in shaping biogeographical patterns. However, much of what we know about plant tolerance to abiotic stresses is based on studies of just a few plant species, most notably the model species *Arabidopsis thaliana*. In this study we examine natural variation in the stress responses of five diverse *Boechera* (Brassicaceae) species. *Boechera* plants were exposed to basal and acquired combined heat and high light stress. Plant response to these stresses was evaluated based on chlorophyll fluorescence measurements, induction of leaf chlorosis, and gene expression. Many of the *Boechera* species were more tolerant to heat and high light stress than *A*. *thaliana*. Gene expression data indicates that two important marker genes for stress responses: *APX2* (Ascorbate peroxidase 2) and *HsfA2* (Heat shock transcription factor A2) have distinct species-specific expression patterns. The findings of species-specific responses and tolerance to stress indicate that stress pathways are evolutionarily labile even among closely related species.

## Introduction

This study examined the responses to combined high heat and high light stress among a group of native California plants in the genus *Boechera* [1–3]. The *Boechera* (Brassicaceae) are closely related to the model species *Arabidopsis thaliana* [4,5] and live in a variety of habitats from high elevation mountains, to coastal regions, to deserts [6,7]. This combination of being closely related to the model species *A*. *thaliana* and having undergone natural selection under varying climatic conditions provides a useful system with which to examine plant stress tolerance. In this study we examine the stress responses of five *Boechera* species that are native to California: *B*. *arcuata*, a widespread species is found in low elevations; *B*. *californica*, is found in the chaparral regions of southwestern CA; *B*. *depauperata* is a high elevation species found in the Sierra Nevada mountains; *B*, *johnstonii* is a rare and endangered species found in the mountains of southern CA; and *B*. *perennans*, is found in the deserts and chaparral of southwestern CA. We compare their responses to stress to that of the model species *A*. *thaliana* Col.

Heat stress in plants is well understood, especially in the plant model *Arabidopsis thaliana* [6–8]. High light stress is also well studied in model plants [9]. However, while there have been some study of combined stress there is far less is known about how plants respond to multiple simultaneous stresses than how they respond to single stressors [10–13]. In addition, very little is known about the stress responses of non-model species.

Plant responses to stress are complex, and involve a combination of physiological, cellular, metabolic, and transcriptomic changes [14]. Research on *Arabidopsis thaliana* [15], *Sorghum bicolor* [11] and *Solanum lycoperscion* [16] have found that a combination of stresses produces changes in gene expression and the activation of gene transcripts that were not induced by either stress alone. Therefore while it is useful to characterize the response of plants to stresses individually, the results of single-stress studies do not reflect the impact of combined stress [14]. Importantly these studies also do not fully represent the types of responses seen in nature and it has been proposed that each stress combination be treated and studied as a new stress altogether [17].

Chlorophyll fluorescence (CF) is widely used in physiological studies to monitor photosynthesis [18,19]. Two such measurements of CF are Fv/FM (optimal quantum yield, ratio of variable fluorescence [Fv] to maximal fluorescence [FM]) and YII (effective quantum yield [Y] of photosystem II), and these are used to examine the impact of stress on the activity of PSII (photosystem II). Light energy that is absorbed by the leaf that is not used for photosynthesis and is not dissipated as heat, is re-emitted as CF [9]. A decrease in CF is an indication of damage to the chlorophyll molecule and that photosynthetic capacity has been reduced. Chlorophyll fluorescence can be measured by exposing a leaf surface to a specific wavelength of light and comparing that to the amount of light that is re-emitted at a longer wavelength [19]. YII measurements of CF do not require dark-adapted leaves and thus are appropriate for the study of stress-induced changes in CF [20].

The expression patterns of key stress-induced genes can provide important information on the activity of stress pathways and here we examined the expression of three of these, described below. Ascorbate peroxidase, (*APX2*, AT3G09640), is a hydrogen peroxide scavenging enzyme that is found in the cytosol of plant cells. *APX2* is an important part of the plant response to reactive oxygen species (ROS) [21–23]. The expression of *APX2* is often used as a marker of the oxidative stress response in plants. Early light Inducible proteins (ELIPs) are members of the light-harvesting complex protein superfamily [24,25]. Early light-inducible protein 2, *ELIP2* (AT3G22840) is known to be expressed in *Arabidopsis* under high light and is thought to bind to chlorophyll a, lutein, and carotenoids, and to affect the production of photosynthetic pigments as well as absorb sunlight [21,25]. Heat shock transcription factor A2 (HsfA2) (AT2G26150) is a key heat shock transcription factor that turns on the expression of the heat-inducible proteins such as the Heat Shock Proteins (HSPs) during heat and other stresses [26,27].

In this study we examined plant responses to combined heat and high light stress. We have found extensive variation in plant tolerance and response to heat and high light stress. Variation was found both among the five *Boechera* species studied and between *Boechera* and *A*. *thaliana*. Interestingly we found that tolerance to stress was not strictly related to habitat, i.e. desert plants are not the most tolerant to combined heat and high light stress. Our results indicate that responses to combined stress are complex even among closely related species. Further, our findings suggest that studies of natural variation to combined stress responses will both inform studies that seek to dissect plant response pathways and studies that seek to understand plant biogeography and conservation.

## Materials and Methods

### Germination of seeds and growth of plants

Seed germination and growth methods were based on standard *A*. *thaliana* protocols (https://abrc.osu.edu/seed-handling) that were optimized for *Boechera*. Prior to being plated on MS agar medium, seeds were sterilized using ethanol, bleach, and TRITONX100. Seeds are then plated (at 10–15 seeds per plate) and cold treated in the dark at 4°C. *Boechera depauperata* seeds were cold treated for three weeks and all other species, including *A*. *thaliana*, were cold treated for 3–4 days. The plates were then moved to Percival E-36 growth chambers and plants were grown for 7–10 days at 22°C with a light intensity of 150 μmol m^-2^ s^-1^. During each stress treatment (described below), 4–5 of these plates were placed in the chamber at once, for 1 replicate of ~50 seedlings, each of which served as a biological replicate. All stress treatments were repeated at least once, for a minimum of 2 experiments per stress treatment, with a total of ~100 seedlings/biological replicates. Table 1 provides seed source and locality information for the species studied.

**Table 1:** Seed Sources for *Arabidopsis thaliana* and *Boechera* species.

Species	Voucher or Accession Information	GPS Coordinates	County
*A*. *thaliana*	Col-0 (ABRC)	44.053889, 3.6925	N/A
*B*. *arcuata*	TPF-0097-08-W	34.239026, -118.359064	Los Angeles
*B*. *californica*	SDNHM 156085	32.8117, -116.674	San Diego
*B*. *depauperata*	CCH YM-YOSE224299	37.648, -119.403	Merced
*B*. *johnstonii*	UCR 202616	33.814723, -116.679189	Riverside
*B*. *perennans*	*SDNHM 193153*	*32*.*701*, *-116*.*128*	San Diego

The names and accession information is provided for all species studied. The ABRC accession number is provided for *A*. *thaliana* and for all other species the voucher number or accession is provided. TPF is the Theodore Payne Foundation, SDNHM is the San Diego Natural History Museum, YM is the Yosemite National Park Herbarium and UCR is the University of California at Riverside Herbarium.

### Stress Experimental Conditions

**Fig 1:**
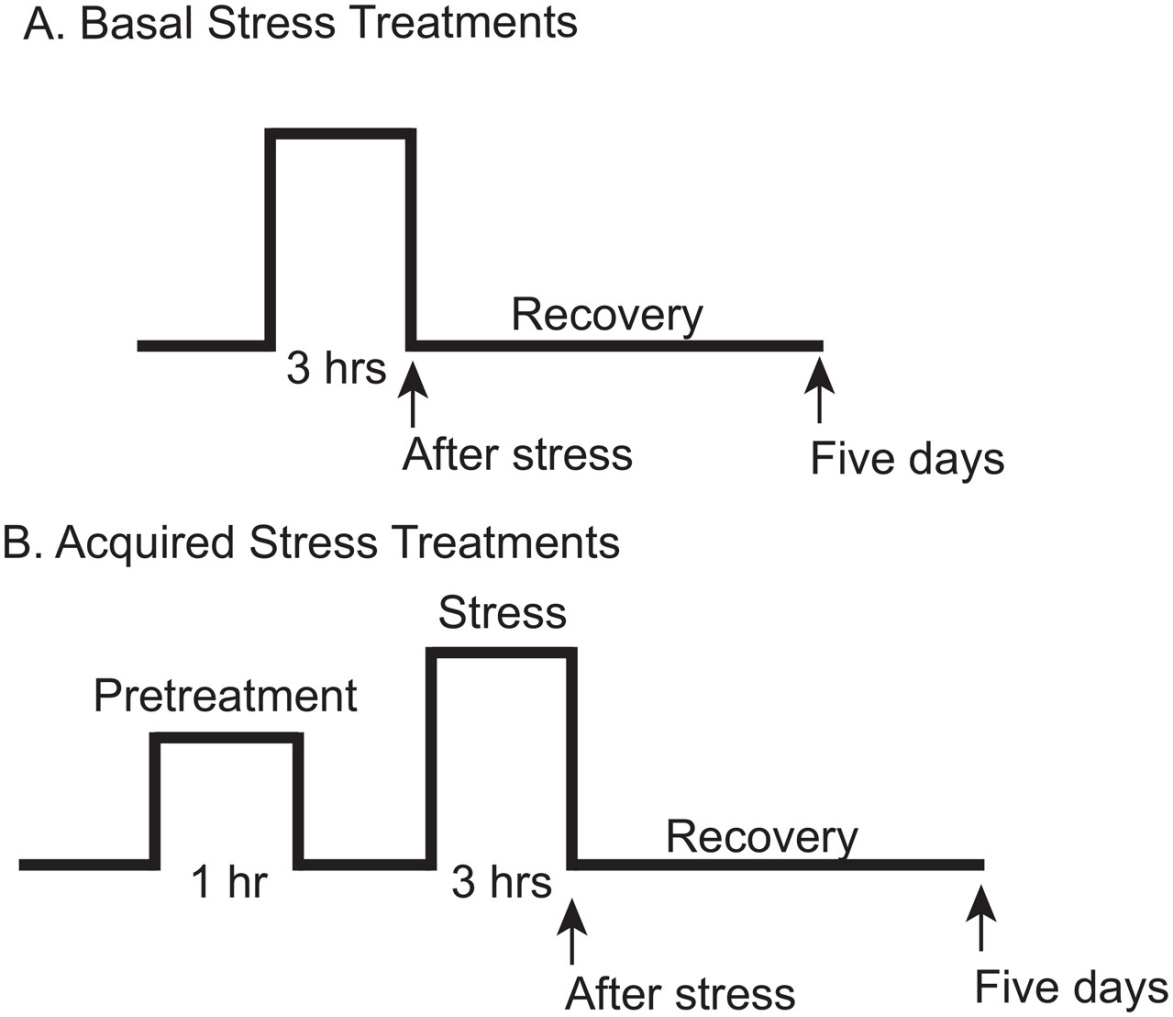
Experimental Design. Arrows indicate when chlorophyll fluorescence measurements were taken. Leaf damage scores were taken after the five-day recovery period. Plants were grown at control conditions (22°C with 150 μmol m^-2^ s^-1^) until exposed to stress treatments. A. Basal stress treatment. Plants were moved from control growth conditions to the stress treatments: 1200 μmol m^-2^ s^-1^ and one the following temperatures 38°C, 41°C, 43°C, 45°C. B. Acquired stress. Plants were first given a pretreatment of either heat only: 38°C, or combined heat and high light (1200 μmol m^-2^ s^-1^): 38°C/HL followed by stress treatments of 41°C, 43°C, 45°C.

At 7–10 days old seedlings were exposed to either basal or acquired stress treatments, see Fig 1 [28]. Basal stress experiments do not contain a pre-treatment. In these experiments plants were moved directly from control conditions to stress treatments for three hours (Fig 1A). Acquired treatments included a one hour pretreatment followed immediately by a recovery period and then followed immediately by a more intense stress for three hours (a total of 5 hours in the same day) (Fig 1B). In the high light stress treatments plants were exposed to a light intensity of 1200 μmol m^-2^ s^-1^. Heat pre-treatments were conducted at 38°C. The heat stress treatments included 38°C, 41°C, 43°C and 45°C treatments. All stress treatments were conducted for three hours. All recovery periods were at control conditions of 22°C and 150 μmol m^-2^ s^-1^. Each experiment was replicated. The control light condition is 150 μmol m^-2^ s^-1^. The abbreviation HL is used for the high light or light stress condition: 1200 μmol m^-2^ s^-1^. We will use the notation of “/” to indicate a combined stress treatment, i.e. 38°C/1200 μmol m^-2^ s^-1^ or, 38°C/HL and a “+” to indicate an acquired treatment, with the stress indicated before the “+” as the pretreatment, and after the “+” as the stress treatment, i.e. 38°C/HL +43°C.

### Chlorophyll fluorescence

Photosynthetic measurements of chlorophyll fluorescence (YII) were taken using a JR PAM Chlorophyll Fluorometer.[29] This was performed on seedlings before stress treatment, immediately following stress treatments, and 5 days after stress treatments to assess plant recovery. We did not collect dark adapted (Fv/Fm) measurements because we wished to collect information immediately after the stress was imposed. The measure YII has been extensively used in stress studies [18,19].

### Leaf chlorosis

**Fig 2:**
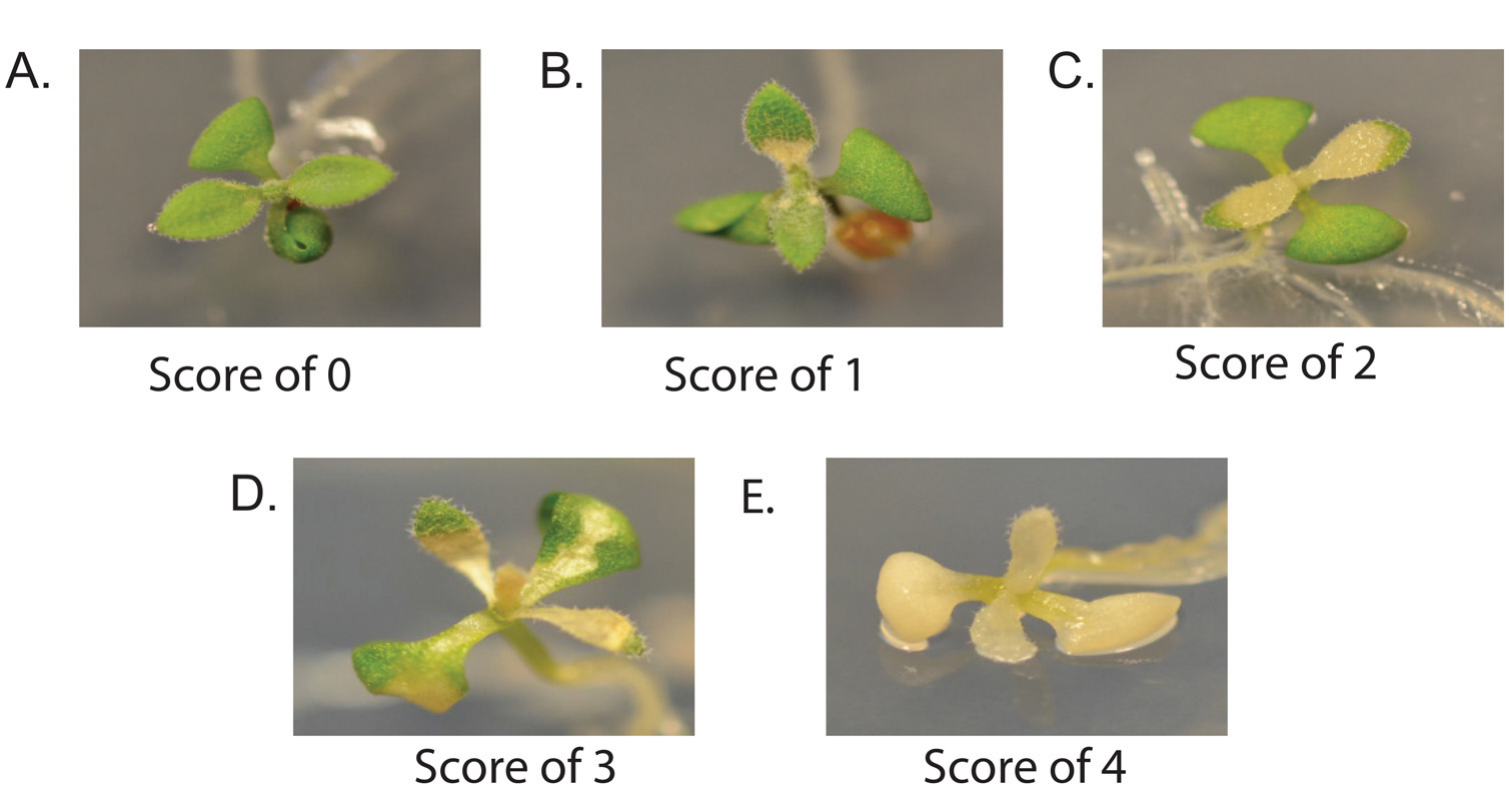
Leaf Damage Scores. A. No signs of stress, plant completely green, a score of 0. B. A few chlorotic spots on plant, appears speckled, a score of 1. C. Less than half of plant chlorotic, a score of 2. D. More than half of plant chlorotic, a score of 3. E. Plant is entirely chlorotic, a score of 4.

After the five-day recovery period each seedling was also given a leaf damage (leaf chlorosis) score to assess leaf damage and survival. This score ranged from zero (no signs of chlorosis; a completely healthy plant) to 4 (a completely dead; chlorotic plant). This gives an overall response to stress of the plants relative to each other. Fig 2 shows examples of each score. An evaluation and survey of thermotolerance scores is discussed in Yeh *et al*. and diagnosis of stress damage using visible foliage symptoms is discussed in Vollenweider *et al*. [30].

### Quantitative PCR analysis of Gene Expression

Leaf tissue was flash frozen in liquid nitrogen and ground to a fine powder immediately after stress. Total RNA was isolated using Ambion RNAqueous. The total RNA was quality checked and then used to generate cDNA. cDNA was synthesized using High Capacity cDNA Reverse Transcription kit by Invitrogen (Life Technologies). Three reference genes were used: EVFP (At3g16270) SAND (At2g28390) and Act2 (At3g18780) to evaluate gene expression changes. Reference genes were chosen using a literature review and GENEVESTIGATOR Plant Biology Version [31,32]. Primers for both reference genes and genes of study are listed in Table 2 and were designed using “PrimerQuest” feature on Integrated DNA Technologies (IDT) using the *A*. *thaliana* TAIR CDS sequences. Sequences were verified experimentally using PCR for *Boechera* species. This was done to determine that there were no nucleotide substitutions among species that would influence qPCR primer efficiency. All primer efficiencies where within good quality range of 90–120 as determined using the method outlined in Yuan *et al*., 2008 [33]. Gene expression changes were calculated using the ΔΔCq method [34].

**Table 2:** Primer sequences for qPCR.

Gene	TAIR Gene Identifier		Primer Sequence 5'- 3'
*EVFP*	At3g16270	F	CAGGTGCCCTCTAATGATAATAAA
*EVFP*	At3g16270	R	CTCATTCTTCTTTGGCAAATGG
*SAND*	At2g28390	F	GATGACTTGCTTCTACTCTCAAA
*SAND*	At2g28390	R	GTTGTATCTTGGTAGGCAGATT
*ACT2*	At3g18780	F	CCTTACCGAGAGAGGTTACATGTT
*ACT2*	At3g18780	R	CCTGCTCGTAGTCAACAGCAACAA
*APX2*	At3g09640	F	CATCCTGGTAGACAGGACAAAG
*APX2*	At3g09640	R	CCATCCGACCAAACACATCT
*ELIP2*	At4g14690	F	ATCAACGGGAGACTAGCAATG
*ELIP2*	At4g14690	R	CGTCAGAGATCTGAGCAAACA
*HSFA2*	AT2G26150	F	CAAGTTTCATTCGTCAGCTCAATAC
*HSFA2*	AT2G26150	R	GAGATGCTTCTGTCCTGCTAAA

Gene name abbreviations are as follows: *EVFP*: ENTH/VHS family protein; *SAND*; SAND family protein. *ACT2*: ACTIN2; APX2: ACORBATE PEROXIDASE2; *ELIP2*: EARLY LIGHT PROTEIN2; *HSFA2*: HEAT SHOCK TRANSCRIPTION FACTORA2

### Statistical Methods

All experiments were independently repeated at least two times. Statistical testing was performed in SYSTAT version 12.0 [35]. In all cases, a p-value of less than 0.05 was used to determine statistical significance. Two-sample Student’s t-tests were performed to determine if the means of two treatments differed significantly. A paired t-test was used in some cases to compare averages of the same individuals (in this case, seedlings) after an amount of time has passed. Analysis of Variance (ANOVA) was used to compare the means of three or more groups. Finally, Tukey's Honestly-Significant-Difference Test was used to compare pairs of means to each other. If an ANOVA test was determined to be significant, then this test was performed.

## Results

In order to examine the responses of *Boechera* species to combined high heat and high light stress, seedlings were exposed to a series of basal and acquired treatments of combined heat and high light stress. The responses of seedlings were monitored by examining stress-induced damage to leaves, the impact of stress of chlorophyll fluorescence, and the induction of a set of key genes that indicate the activity of different stress-induced pathways.

### Chlorophyll fluorescence does not vary across species under control conditions

**Fig 3:**
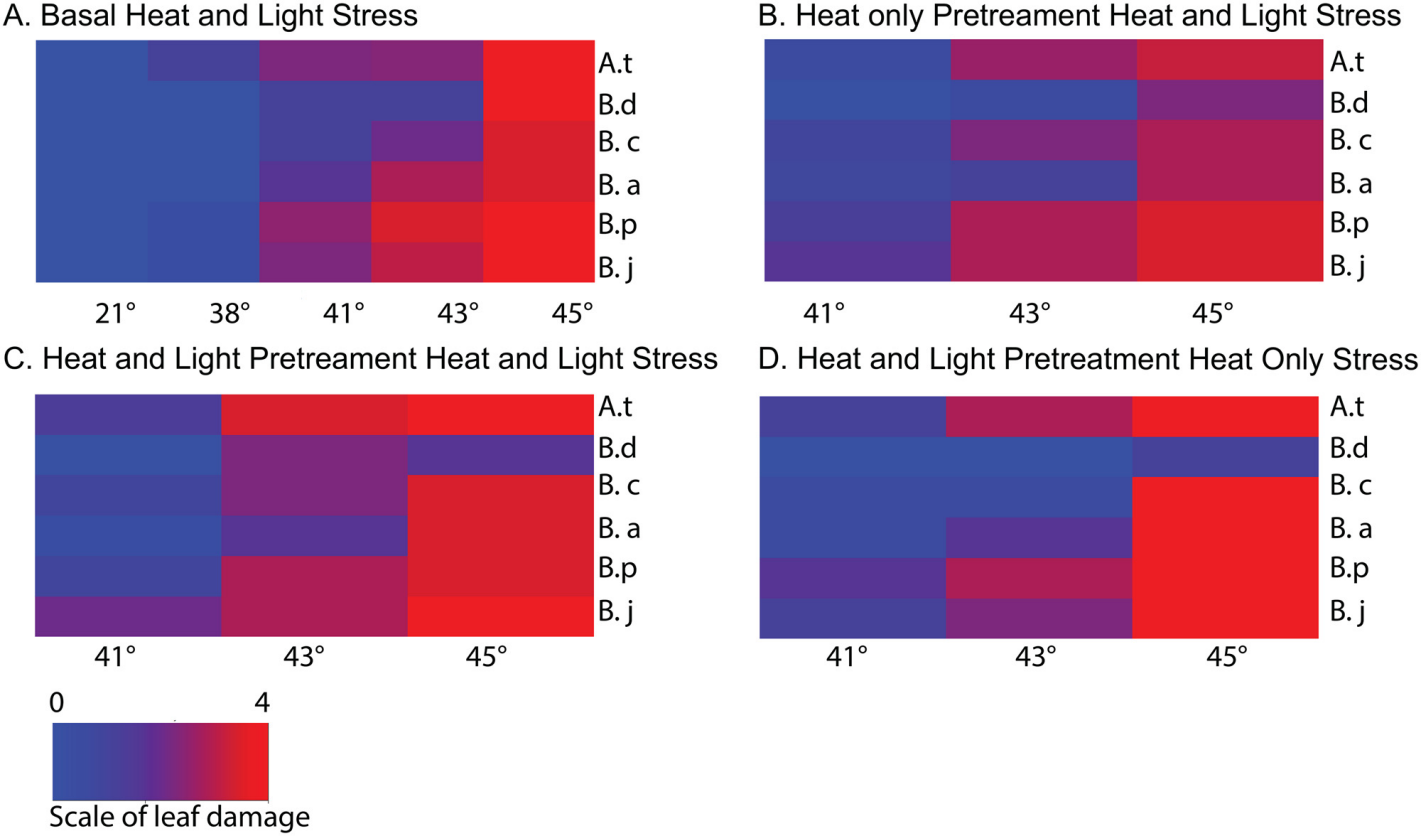
Stress-induced damage to leaves. Heat maps of leaf scores after combined heat and high light treatments. Scores ranged from 0: blue, indicating no damage to 4:red, leaves are completely chlorotic. Species abbreviations are: A.t: *A*. *thaliana*, B.a: *B*. *arcuata*, B.c: *B*. *californica*, B.d: *B*. *depauperata*, B.j: *B*. *johnstonii*, B.p: *B*. *perennans*. A.) Leaf damage scores after a basal combined heat and high light (1200 μmol m^-2^ s^-1^) stress: 38°, 41°, 43°, or 45°C. B.) Leaf damage scores after acquired treatments with a heat pretreatment (38°C) and combined heat and high light (1200 μmol m^-2^ s^-1^) stress treatments of 41°C, 43°C or 45°C. C.) Leaf damage scores after acquired treatment of a combined (38°C/1200 μmol m^-2^ s^-1^) pretreatment and a combined heat and high light (1200 μ μmol m^-2^ s^-1^) stress treatment: 41°C, 43°C or 45°C. D.) Leaf damage scores after acquired treatment of a combined (38°C/1200 μmol m^-2^ s^-1^) pretreatment and a heat only stress: 41°C, 43°C or 45° at low light (150 μmol m^-2^ s^-1^). Statistical analysis using ANOVA reveals that for all treatments (A-D) there are significant (at the 0.05 level) differences among species in the level of induced leaf damage.

Before conducting the stress experiments we first examined the levels of chlorophyll fluorescence among the five different *Boechera* species, as well as *A*. *thaliana* (Col-0). To determine that the plants do not vary in their untreated chlorophyll fluorescence values we examined the YII values of all species at 22°C and control light levels of 150 μmol m^-2^ s^-1^. Under these conditions the chlorophyll fluorescence for each species is in the 0.7–0.8 range, see Fig 3A. The values for chlorophyll fluorescence 5 days later are similar and remain in the 0.7–0.8 range.

### Stress induced leaf damage varies across species

Analysis of leaf damage scores taken five days after stress treatments shown in Fig 3 indicates that the *Boechera* species vary in their tolerance to both basal and acquired stress. In Fig 3A we see the impact of basal combined heat and high light stress on seedling leaves. It is clear that the highest stress (45°C/HL) causes almost complete chlorosis is all species (Fig 3D) and that high light at 22°C causes very little damage (Fig 3A). Variation in species responses is evident at 38°C, 41°C and 43°C (Fig 3A). Of all the species *B*. *depauperata* has the least leaf damage after a basal combined stress of 43°C/HL (Fig 3A). When a heat pretreatment (38°C) is provided before the combined heat and high light stress we see protection (lower leaf damage scores) (Fig 3B) for all species. *B*. *depauperata* has the least damage after the 45°C treatment (Fig 3B). When a combined heat and high light pretreatment proceeded a combined treatment (Fig 3C) leaf damage scores were higher (i.e. more leaf damage). Examination of this data indicates that only *B*. *depauperata* displays tolerance to the highest stress of 45°C/μmol m-2 s-1after a combined pretreatment (Fig 3C). A similar pattern is seen in Fig 3D. In this experiment plants were given a combined pretreatment and a heat only stress. Again *B*. *depauperata* was the most tolerant. It is interesting that across the four different stress treatments the model species *A*. *thaliana* was among the least tolerant species (Fig 3).

### Chlorophyll fluorescence measurements indicate that there are species differences in their ability to protect PSII under heat and high light stress

**Fig 4:**
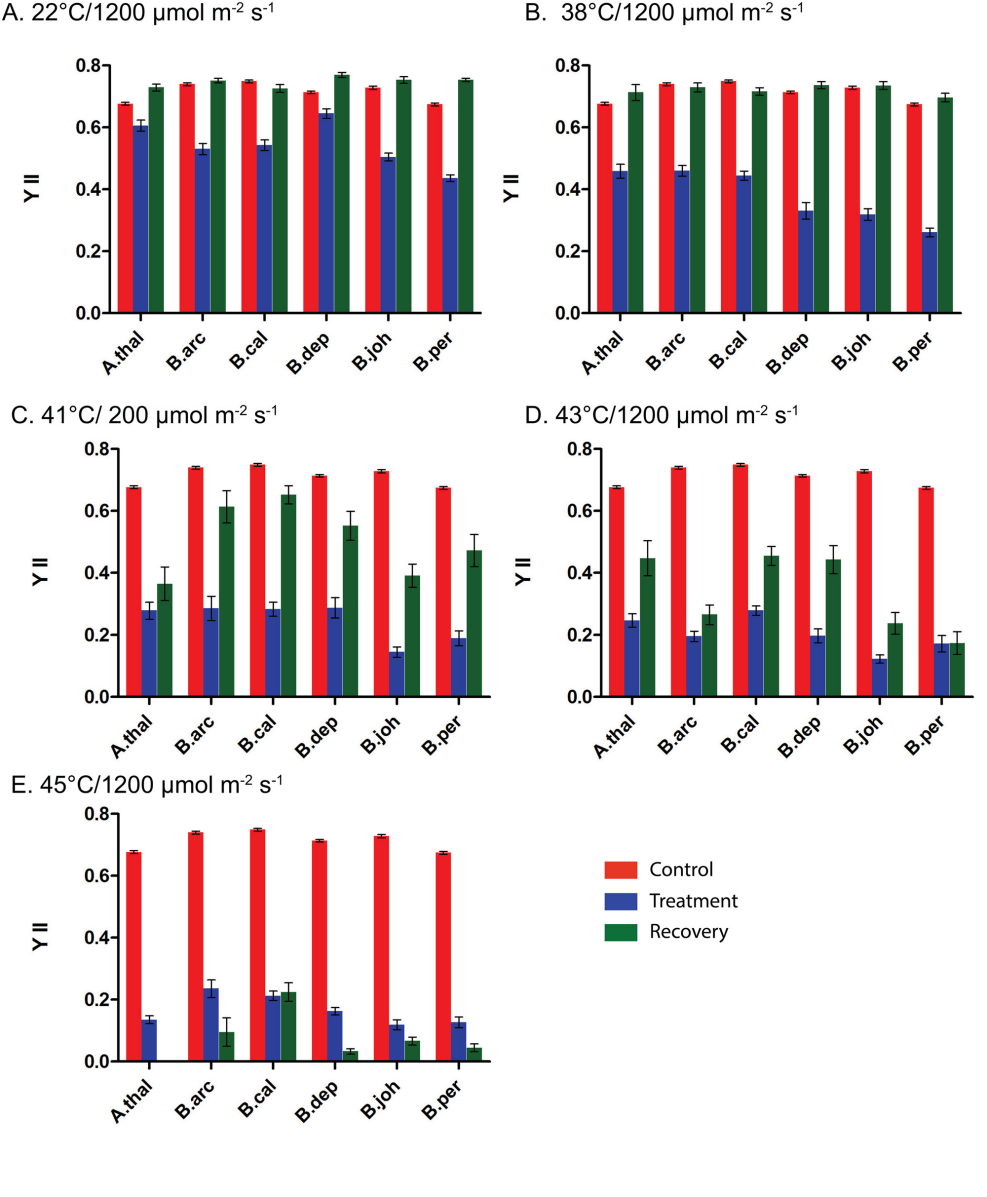
Impact of basal combined high light and high heat stress on chlorophyll fluorescence. A.) 22°C/1200 μmol m^-2^ s^-1^. B.) 38°C/1200 μmol m^-2^ s^-1^. C.) 41°C/1200 μmol m^-2^ s^-1^. D.) 43°C/1200 μmol m^-2^ s^-1^. E.) 45°C/1200 μmol m^-2^ s^-1^. Control measurements are in red. Measurements taken immediately after stress treatments are in blue and measurements taken after a five day recovery period are in green. Species abbreviations are: Athal: *A*. *thaliana*; Barc: *B*. *arcuata*; Bcal: *B*. *californica*; Bdep: *B*. *depauperata*; Bjoh: *B*. *johnstonii*; Bper: *B*. *perennans*.

The data presented in Fig 4A indicates that all species experience a reduction in YII immediately after exposure to high light (HL). However, there are clear differences among the species, with *B*. *perennans* experiencing the largest reduction and *B*. *depauperata* the smallest reduction in YII after HL stress. However, all species completely recover from this stress, i.e. YII values after five days are at or above control values. When heat stress is added to light stress (Fig 4B) there is an even larger reduction in YII immediately after stress; but again all species recover back to control levels five days after stress. The impact of combined stress is very different when high light stress is combined with exposure to higher heat stress temperatures. The combined stresses of 41°C/HL (Fig 4C) and 43°C/HL (Fig 4D) significantly reduce YII compared to control values. While there is some increase in YII five days after the stress treatments, none of the species fully recover, i.e. all YII values at five days are significantly below that of control YII values. However, we can see clear differences among the species in the ability to recover from combined heat and high light stress. Close examination of the data presented in Fig 3C and 3D reveals that *B*. *arcuata*, *B*. *johnstonii* and *B*. *perennans* all have much lower YII values after the five day recovery period than does *A*. *thaliana*, *B*, *californica* and *B*. *depauperata*. Not unsurprisingly, the combined 45°C/HL treatment has the largest impact on YII values both immediately after stress and after a five day recovery period.

### Pretreatment with high light and/or heat alter species responses to later stress treatments

**Fig 5:**
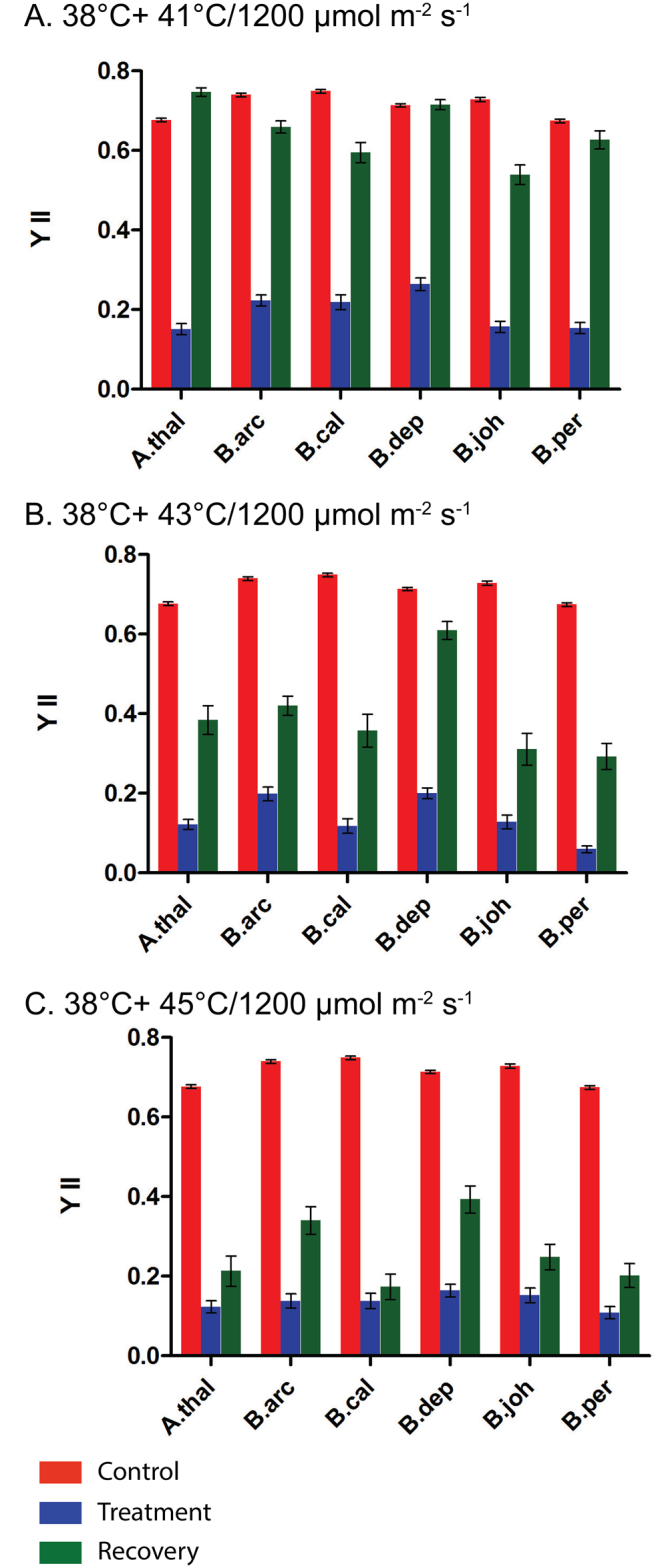
Impact of heat pretreatment of 38°C followed by combined heat and high light treatments on chlorophyll fluorescence. A.) 38°C+ 41°C/1200μmol m^-2^ s^-1^, B.) 38°C+ 43°C/1200μmol m^-2^ s^-1^ C.) 38°C+ 45°C/1200μmol m^-2^ s^-1^. Control measurements are in red. Measurements taken immediately after stress treatments are in blue and measurements taken after a five day recovery period are in green. Species abbreviations are the same as Fig 4.

It is well established that heat pretreatments (38°C) provide protection against later heat stress. We wished to determine the impact of heat and combined heat and HL pretreatments on the ability of *A*. *thaliana* and *Boechera* species to protect PSII during combined heat and HL stress. In Fig 5 we present the impact of a pretreatment of 38°C on YII after combined heat and HL stress. It is clear that a heat pretreatment does provide some protection to PSII when plants are exposed to combined heat and high light (Figs 4 and 5). When the data presented in Fig 4C–4E is compared to that found in Fig 5A–5C it is evident that the YII values are higher in Fig 4 for the same stress treatments, i.e., Fig 1C 41°C/HL with no pretreatment compared to Fig 5A 41°C/HL with a 38°C pretreatment (38°C+ 41°C/HL). This indicates that a heat pre-treatment is to some extent protective against a combined heat and light stress.

Another question we sought to answer was: do all the species respond in the same way to combined stresses? Close analysis of the data presented in Fig 5 suggests that there are differences among the species in their stress response. In Fig 5A the YII values taken immediately after stress and after a five day recovery period are presented for a combined 41°C/HL stress. It is evident that this stress does reduce YII immediately after stress; however, most species can recover and have YII values close to that of control. The two exceptions are *B*. *californica* and *B*. *johnstonii*. Neither of these species was able to completely recover from this stress. As the temperature of the combined heat stress and HL treatment increases the ability of the plants to repair PSII continues to decrease (Fig 5B and 5C). Notably *B*. *arcuata* and *B*. *depauperata* are both able to protect PSII during combined heat and HL stress to a greater extent than are the other species. Both of these species have YII values five days after stress that are much higher than that of the other species. This indicates clear differences in tolerance to stress among the species studied. These findings also indicate significant differences in how each species responds to different stress treatments. For example, *B*. *depauperata* displays a higher ability to recover from basal combined heat and high lights than does *B*. *arcuata* but when given a heat pretreatment both species are able to recover from combined heat and high light stress.

**Fig 6:**
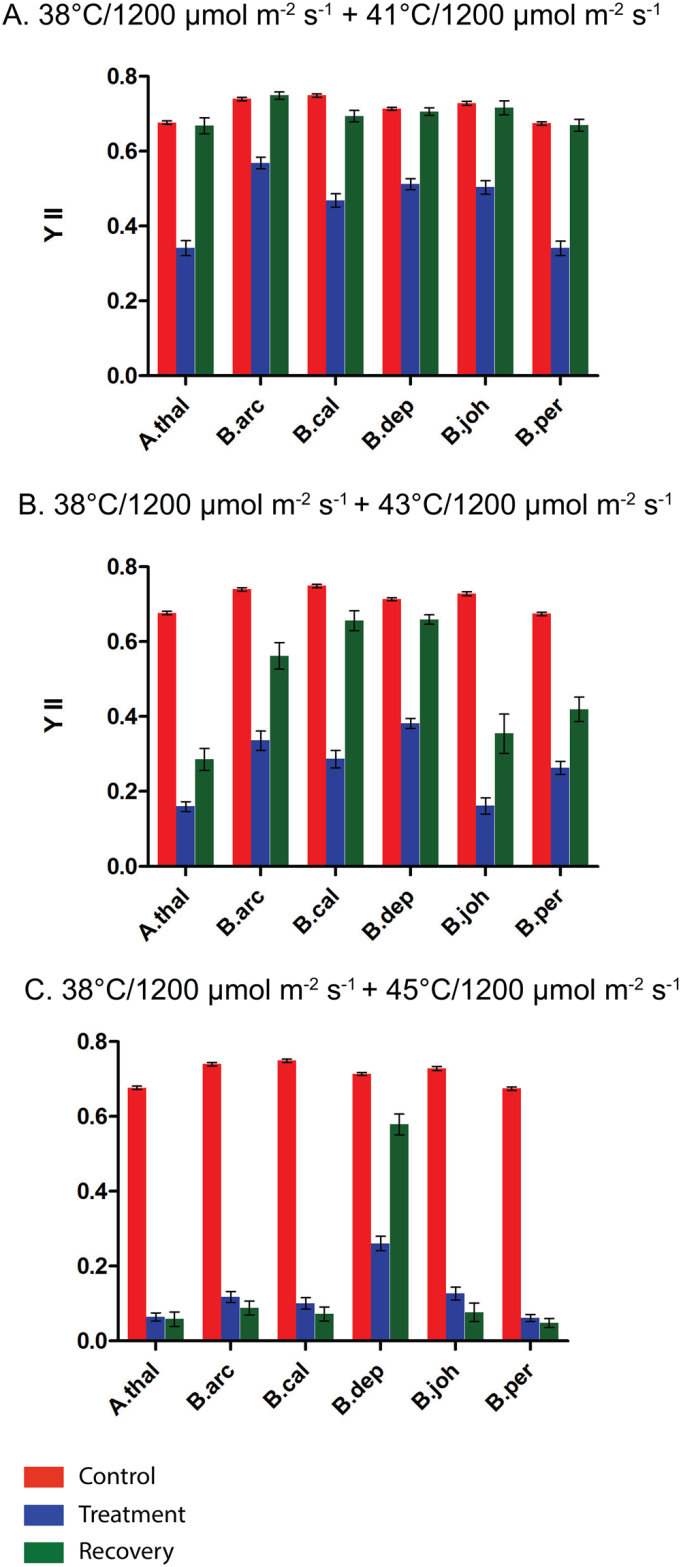
Impact of a combined pretreatment of 38°C and high light followed by combined heat and high light treatments on chlorophyll fluorescence. A.) 38°C/1200μmol m^-2^ s^-1^ +41°C/1200μmol m^-2^ s^-1^, B.) 1200μmol m^-2^ s^-1^ +43°C/1200μmol m^-2^ s^-1^, C.) 38°C/1200μmol m^-2^ s^-1^ +45°C/1200μmol m^-2^ s^-1^. Control measurements are in red. Measurements taken immediately after stress treatments are in blue and measurements taken after a five day recovery period are in green. Species abbreviations are the same as Fig 4

Our data presented in Fig 5 indicates that a heat-pretreatment can provide protection against combined heat and HL stress. We next examined the impact of combined heat and HL pretreatment on combined heat and HL stress. We exposed plants to a pre-treatment of 38°C/HL and then followed with exposures to combined heat and HL. The YII values taken immediately after stress and after a five-day recovery indicate that the combined pretreatment is not significantly more protective than a heat-alone pretreatment (Fig 6). In fact, as the temperature of the stress treatment increases i.e., 43°C/HL (Fig 4B) and 45°C/HL, the YII values are lower for the combined pretreatment plants than they are for the heat-only pretreatments (Fig 5). The data presented in Fig 6 suggests that none of the species are able to completely recover after a combined treatment of 43°C/HL or 45°C/HL. Once again there is evidence of clear species differences in response to stress. The highest levels of YII recovery after the 41°C/HL treatment (Fig 6A) was seen in *B*. *arcuata*, *B*. *depauperata* and *B*. *perennans*. After the 43°C/HL treatment (Fig 6B) *B*. *arcuata* had the highest recovery while *A*. *thaliana*, and *B*. *johnstonii* had the lowest recovery. After the highest level of combined heat and HL stress /tested here (45°C/HL), only *B*. *depauperata* had significant levels of YII recovery (Fig 6C).

**Fig 7:**
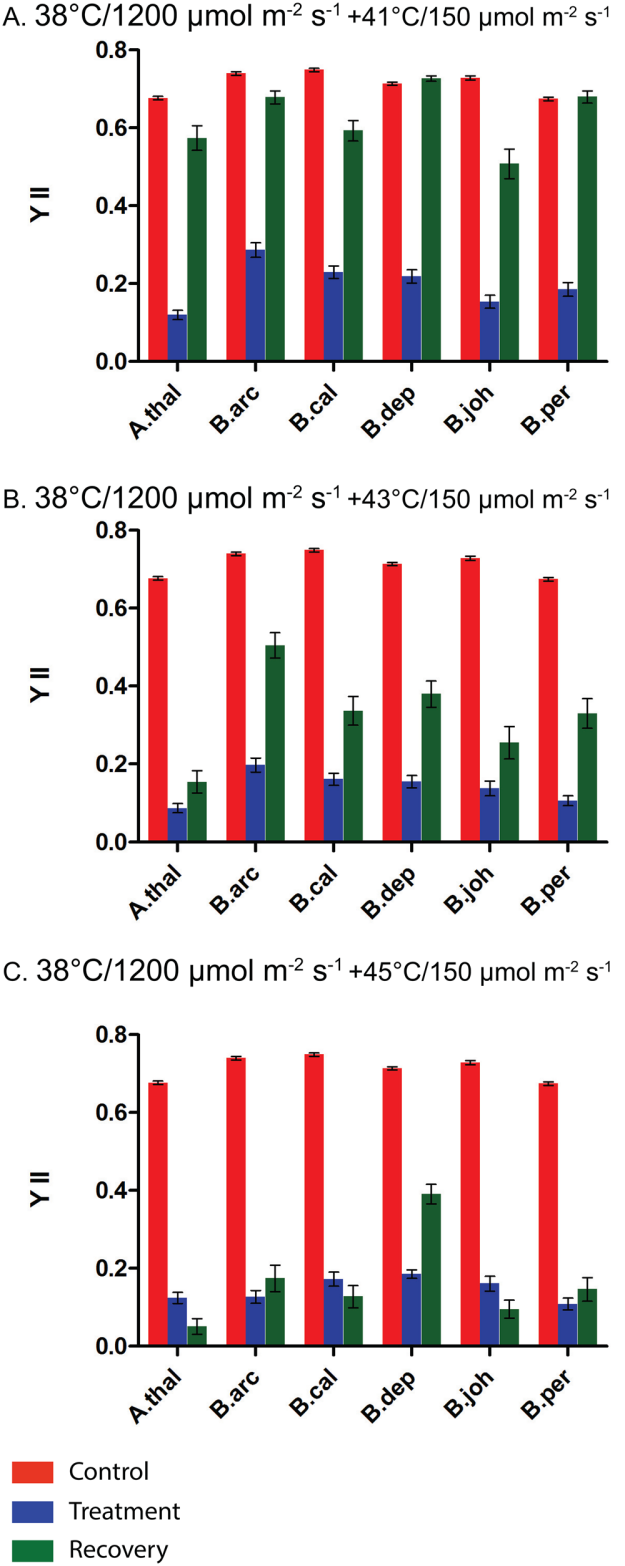
Impact of a combined pretreatment of 38°C and high light followed by a heat only treatment on measurements of YII. A.) 38°C/1200 μmol m^-2^ s^-1^ +41°C/150 μmol m^-2^ s^-1^, B.) 38°C/1200 μmol m^-2^ s^-1^ +43°C/150 μmol m^-2^ s^-1^ C.) 38°C/1200 μmol m^-2^ s^-1^ +45°C/150 μmol m^-2^ s^-1^. Control measurements are in red. Measurements taken immediately after stress treatments are in blue and measurements taken after a five day recovery period are in green. Species abbreviations are the same as Fig 4

It is well established that a heat-pretreatment at 38°C protects against later heat stress. In order to determine if a combined heat and HL stress would provide protection against heat stress we exposed plants to the combined heat and HL pretreatment (38°C/HL) and then challenged the plants with heat stress only. The results of these experiments (Fig 7) shows the five *Boechera* species and *A*. *thaliana* vary in their responses to this type of stress. All species were able to recover to control or near control YII levels after the 38°C/HL+ 41°C/control light (Fig 7A). However, after the 38°C/HL+ 43°C/control light stress none of the species recovered to control YII levels. Again it is notable that there is considerable variation among the species after this treatment. *B*. *arcuata*, *B*. *californica* and *B*. *depauperata* had the highest YII values after the five-day recovery period, and *A*. *thaliana*, *B*. *johnstonii* and *B*. *perennans* had the lowest. After the 38°C/HL+ 45°C/control light treatment (Fig 7C) all but *B*. *depauperata* had very low YII values both immediately after stress and after the five day recovery period (Fig 7C). From the data presented in Fig 7C it is clear that *B*. *depauperata* had the highest YII values immediately after the 38°C/HL+ 45°C/control light stress and demonstrated the highest ability to recover after this stress.

### APX2 is induced in all species by combined heat and high light stress

**Fig 8:**
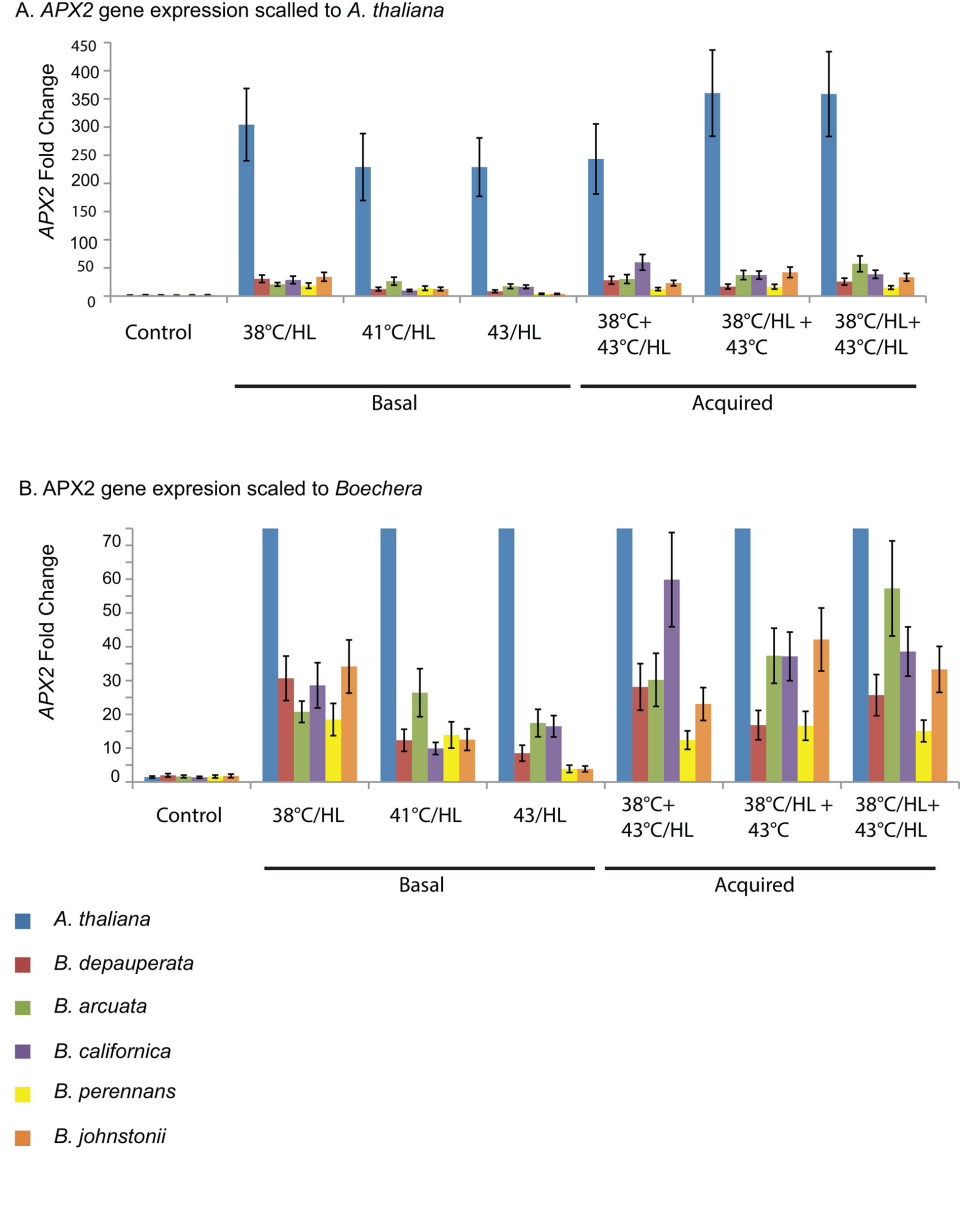
Stress induced expression of *ASCORBATE PEROXIDASE 2 (APX2)*. Gene expression change calculated using the ΔΔCq method for *APX2* compared to reference genes and to control expression. Basal combined treatment include 38°C, 41°C and 43°C at high light (HL:1200 umols m^-2^s^-1^). Acquired treatments include combined heat and high light (HL) pretreatments and heat only pretreatments (38°C). A.) *APX2* gene expression scaled to illustrate *A*. *thaliana* expression levels. B.) *APX2* gene expression scaled to illustrate *Boechera* expression levels.

Ascorbate peroxidase is important in the ROS scavenging network and is known to be upregulated by heat and oxidative stresses [36]. Examination of *APX2* expression patterns provides information on the status of the oxidative stress response within cells. Here we report that when exposed to combined heat and high light stress all *Boechera* species express *APX2*. There are some interesting differences in expression pattern among the species studied. It is clear that *A*. *thaliana* has very high expression levels across all treatments (Fig 8A). When the scale on the fold changes is adjusted (Fig 8B) we see that while the *Boechera* all have lower overall fold changes than does *A*. *thaliana*, all *Boechera* species show expression of *APX2* during the stress treatments.

There are species differences in *APX2* expression patterns. *A*. *thaliana* has the highest *APX2* expression levels after a combined 38°C/HL pretreatment and a 43°C stress as well as after a combined 38°C/HL pretreatment and a 43°C/HL stress (Fig 8A). The lowest levels of *APX2* expression in *A*. *thaliana* were seen after the basal combined heat and HL stresses of 41°C/HL and 43°C/HL (Fig 8A). The lowest levels of *APX2* gene expression for the *Boechera* species were also seen after these two stress treatments, 41°C/HL and 43°C/HL (Fig 8B). The highest level of *APX2* gene expression differed among the *Boechera* species. A 38°C pretreatment followed by a 43°C/HL treatment induced the highest gene expression in *B*. *perennans* and a combined 38°C/HL pretreatment followed by a 43°C/HL treatment induced the highest *APX2* expression in *B*. *arcuata* (Fig 8B).

### Species-specific ELIP2 expression patterns

**Fig 9:**
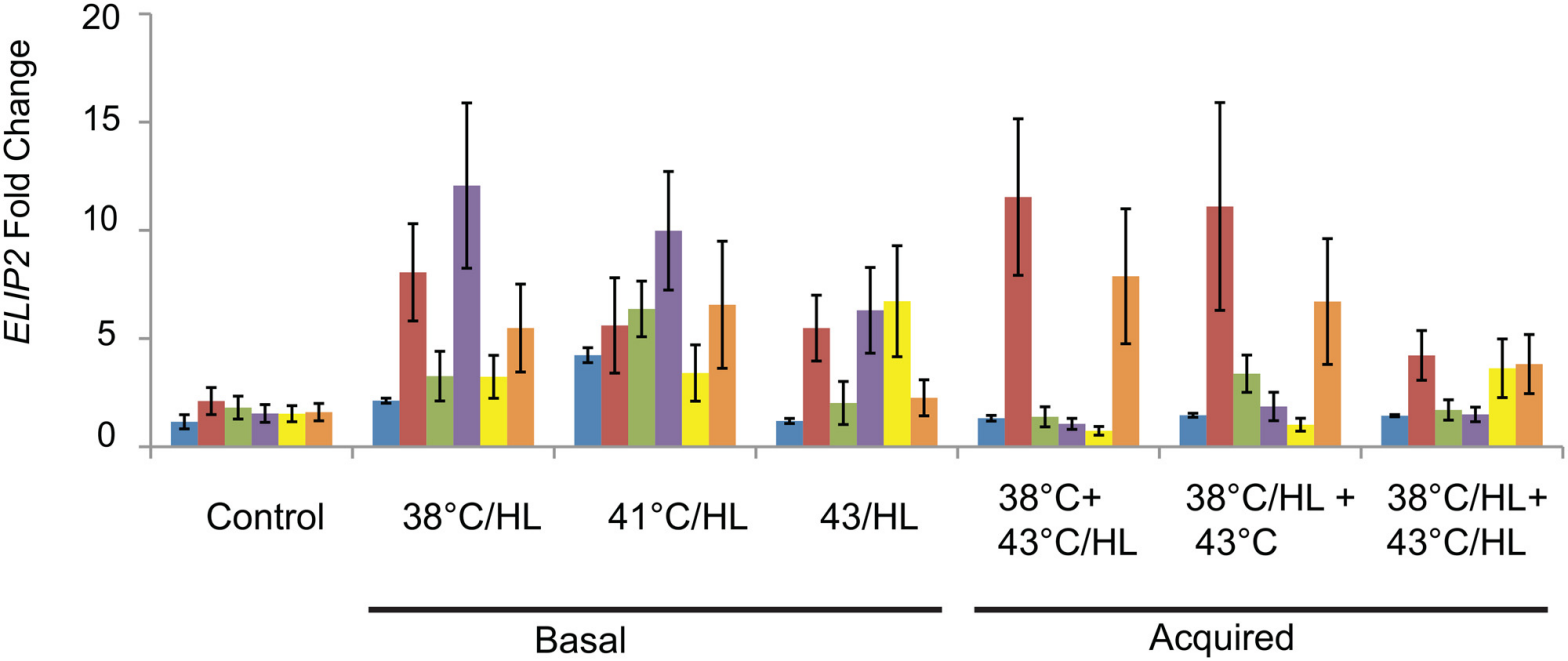
Stress induced expression of *EARLY LIGHT INDUCED PROTEIN 2 (ELIP2)*. Gene expression change calculated using the ΔΔCq method for *ELIP2* compared to reference genes and to control expression. Basal combined treatment include 38°C, 41°C and 43°C at high light (HL: 1200μmol m^-2^ s^-1^). Acquired treatments include combined heat and HL pretreatments 38°C/HL and heat only pretreatments (38°C). The color legend is the same as shown in Fig 8.

The gene expression patterns for *ELIP2* are quite different than that of *APX2*. While it is clear that *ELIP2* is expressed under most treatments, the level of fold change, especially for *A*. *thaliana*, is much lower than that of *APX2*. However, interestingly we see larger differences among species for *ELIP2* expression (Fig 9). It is notable that for some treatments *B*. *depauperata*, the species that is best able to protect PSII during stress has the highest level of *ELIP2* expression. Some species, including *A*. *thaliana*, *B*. *californica*, and *B*. *arcuata* often have quite low ELIP2 expression levels.

### All species express HsfA2 during combined heat and HL stress

**Fig 10:**
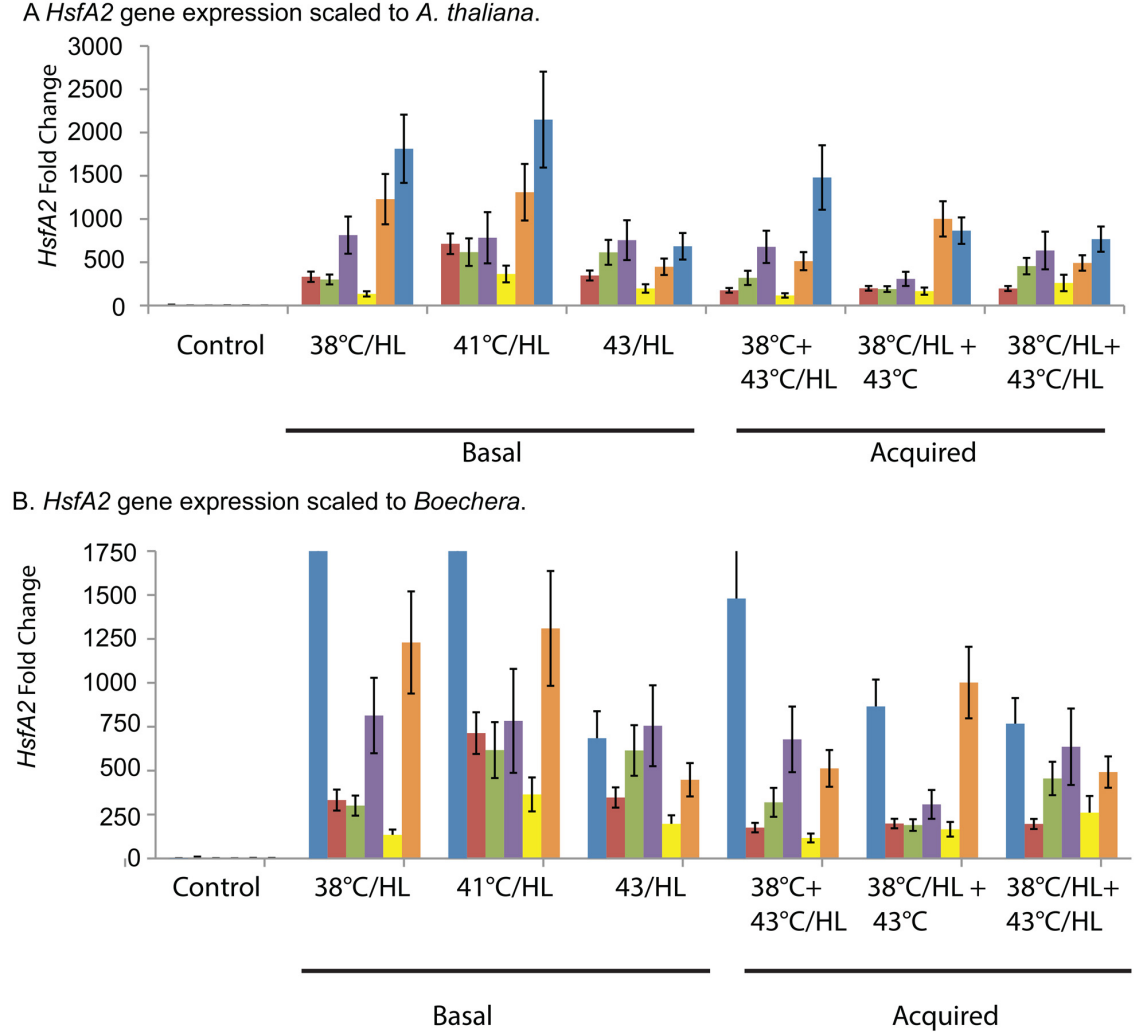
Stress induced expression of *Heat Shock Transcription Factor A2 (HsfA2)*. Gene expression change calculated using the ΔΔCq method for *HSFA2* compared to reference genes and to control expression Basal combined treatment include 38°C, 41°C and 43°C at HL 1200μmol m^-2^ s^-1^ (i.e. 38°C/HL). Acquired treatments include combined heat and high light pretreatments 38°C and 1200μmol m^-2^ s^-1^ (38°C/HL) and heat only pretreatments (38°C). A.) *HsfA2* expression scaled to illustrate *A*. *thaliana* expression levels. B.) *HsfA2* expression levels scaled to illustrate *Boechera* expression levels. The color legend is the same as shown in Fig 8.

*HsfA2* is a well-characterized heat shock transcription factor. It is known to be important in turning on the heat shock proteins during heat stress and has a role in other stresses [26,27]. As expected, all of the species studied have stress-induced expression of *HsfA2* (Fig 9). The data presented here indicate that among the species studied here, *A*. *thaliana* has the highest fold changes (Fig 10). A basal 41°C/HL treatment induced the highest expression of *HsfA2* in *A*. *thaliana*. Among the *Boechera* species *B*. *johnstonii* has the highest fold change in *HsfA2* expression. The basal treatments of 38°C/HL induced the highest expression in *B*. *johnstonii*, a similar pattern to that seen for *A*. *thaliana*.

## Discussion

It is well established that a heat pretreatment can provide plants with acquired thermotolerance [37] [8]. This is accomplished by the induction of genes that allow the plants to tolerate higher temperatures. Much of the work conducted on plant stress has been conducted on just a few model (*A*. *thaliana*) or agriculturally important species [38,39]. Much less is known about variation in stress responses in natural populations. This is despite the fact that stress tolerance is a crucial aspect of plant biology. In a recent study of tolerance to heat stress between two species of *Potamogeton* it was found that both basal and acquired tolerance to heat stress can vary among closely related species [40]. We have been interested in variation in tolerance to abiotic stress among *Boechera* species. It has been previously shown that the induction of the heat shock proteins in the *Boechera* provides added acquired thermotolerance (Halter and Waters. 2012). In this study we examine the more complex stress response to combined heat and HL stress among five *Boechera* species and *A*. *thaliana*. Here we clearly demonstrate the protection provided by a heat pretreatment. The added protection of pretreatment is evident both in the stress induced leaf damage (Fig 3) and in the impact stress has on YII (Figs 4–7). The impact on YII is most evident after the 41°C/HL and 43°C/HL treatments (Fig 4C and 4D and Fig 5A and 5B). When a heat only pretreatment is given it is clear that all the species studied have a higher tolerance to combined heat (41°C) and HL stress (Fig 5A). However, of the six species studied only two (*B*. *arcuata* and *B*. *depauperata*) had YII values close to that of controls after a 43°C/HL treatment after a heat pretreatment (Fig 5B). When the data in Fig 4E is compared to that in Fig 7C it is clear that all species are severely impacted by the higher stress of 45°C/HL but *B*. *arcuata* and *B*. *depauperata* have higher acquired thermotolerance even at these extreme conditions.

A heat pretreatment provides protection against a combined heat and HL stress but it is clear from a comparison of Figs 5 and 6 that a combined heat and HL pretreatment does not provide additional protection against a combined heat and HL stress. With the exception of *B*. *arcuata* and *B*. *depauperata*, YII values are lower after the combined pretreatment followed by a 41°C/HL treatment than after the same treatment and a heat only pretreatment (Figs 5A and 6A). However, even for *B*. *arcuata* and *B*. *depauperata*, more intense stresses (Fig 5B and 5C vs. Fig 6B and 6C) with a combined pretreatment result in greater reductions in YII. The variation among the species responses to these stresses is also illustrated in Fig 7. Here we see that once again at the highest stress 45°C (Fig 7C) only *B*. *depauperata* is able to recover after 5 days.

### Complex species-specific responses to combined heat and HL stress

It has been noted that plant responses to combined stresses cannot be predicted from the responses to individual stresses [11,15,16]. The data presented in this study indicate that it is also not possible to predict species-specific responses patterns to combined stress from their responses to single stresses. It is clear that *B*. *depauperata* is the most tolerant species and that *B*. *johnstonii* is the least tolerant species. However, the responses of the other species to the different combinations of combined heat and HL stress are complex and vary by pretreatment given and the types and of level of stress. For example, as described above *B*. *arcuata* is not tolerant to basal stress (Fig 4) but is significantly more tolerant when a pretreatment is given (Figs 5–7). It is notable that *B*. *californica* is among the most tolerant to basal stress (Fig 4) but is among the least tolerance to combined pretreatments with combined stress (Fig 6). *B*. *perennans* is among the least tolerant to combined basal stress (Fig 4) and a heat pretreatment provides clear protection in this species against the combined 41°C/HL (Fig 5A). *B*. *perennans* is among the least tolerant species to higher heat treatments (Figs 5–7). Further it is interesting to note that no matter what the pretreatment the model species, *A*. *thaliana*, does not recover from treatments that include a 43°C or higher heat stress. These findings suggest complex layers of variation among these species. Johnson *et al*. (2014) have reported that aspects of combined plant stress responses are unique to combined stress [11]. The data here suggests that aspects of combined stress responses are also species-specific. Future work to examine the genome-wide expression patterns of the five *Boechera* species is planned and should shed considerable light on role of pretreatments and stress tolerance in these species.

### Photoinhibition and Photoprotection

Photoinhibition is caused by excess light and is exacerbated by other abiotic stresses such as heat [41–44]. The level of photoinhibition caused by stress is related to the rate of damage to PSII and the rate of repair [41,45,46]. Plant photoprotection mechanisms include ROS scavenging, dissipating light energy within the chloroplast, and chloroplast avoidance [41]. The repair of PSII is a complex process that involves identification of damaged PSII complexes and the synthesis of new PSII complexes [47,48]. Environmental stress increases the presence of ROS in the cell and in addition to the to the direct damage to PSII ROS can generate this increase can also reduce the normal PSII repair process [42]. It is important to note that while ROS is generated during stress all ROS production is not necessarily a symptom of cellular stress or dysfunction because ROS is also an important signaling molecule [49,50]. Feedback de-excitation, or non-photochemical quenching (NPQ), is thought to protect PSII from photoinhibition through the xanthophyll cycle using violaxanthin de-epoxidase (VDE) [51]. Recent studies have suggested that this is a rapid response that converts the light harvesting antenna to heat dissipation (resulting in an immediate decrease in YII as seen in this study), however, high light stress alone did not negatively impact the fitness of *Arabidopsis* mutants deficient in VDE [51]. Rather, a decrease in fitness for the mutants was seen under variable light conditions [51]. These findings are consistent with a lack of change in gene expression for VDE under high light conditions found here in this study (S1 Fig).

A close examination of the YII data presented in this study shows an interesting pattern of both photoinhibition and repair. It is clear that *B*. *depauperata* is the most tolerant species to all the stress treatments given. However, for most treatments this is not due to differences in YII immediately after stress (stress-induced damage to PSII) but rather in the ability to recover after stress. For example when Fig 5B and 5C are examined it is clear that the YII level for all species is quite similar immediately after the 43°C/HL or 45°C/HL stress (YII <0.2) but *B*. *depauperata* and *B*. *arcuata* are able to recover to a greater extent than are the other species. This pattern is also seen in Fig 6C when all species experience a significant decrease in YII after stress (YII = <0.2) but *B*. *depauperata* has the highest recovery (YII = 0.4). In Fig 7C (45°C stress after a 38°C/HL pretreatment) it is clear that *B*. *depauperata* demonstrates both a better capacity to protect PSII (YII>0.25) and to recover PSII activity (YII>0.55) than do the other species. This data clearly suggests species differences in the ability to repair damage to PSII and it raises the question of how this is accomplished.

### Photoprotection: The roles of ROS, APX2 and ELIP2

Two methods of minimizing photoinhibition or achieving photoprotection are ROS scavenging and pigment availability [41,47]. It has been reported that the ELIPS are important in the regulation of chlorophyll accumulation in the chloroplasts [52]. ELIPS are transiently synthesized when there is perturbation of the chlorophyll organization. The ELIPS then reduce the chlorophyll level by inhibiting chlorophyll synthesis. Inhibition of the targeting of ELIPS to the thylakoids is associated with photo-oxidative stress [25]. Tzvetkova-Chevolleau *et al*. 2007 have proposed that the ELIPS are chlorophyll sensors that prevent photo-oxidative stress by modulating chlorophyll synthesis [52]. It is then very interesting that there is higher expression of *ELIP2* in *B*. *depauperata* (Fig 9), the species with the highest ability to recover PSII activity after combined stress. Interestingly *B*. *johnstonii* also has elevated *ELIP2* expression (Fig 9) but it does not have high stress tolerance. Further, we have shown that *A*. *thaliana* has among the lowest *ELIP2* fold changes and also one of the lowest recovery rates. Further research is needed into the role of *ELIP2* in the ability of *B*. *depauperata* to recover from stress.

The gene for ASCORBATE PEROXIDASE 2 (*APX2*) has been shown to be important in plant response to high light and other stresses [22,23,41,53,54]. APX2 is a scavenger of ROS and the induction of *APX2* expression occurs early in HL response [41]. The expression of *APX2* is a strong indicator that the cell is experiencing stress and that ROS are present. The heat shock transcription factor *HsfA2* is known to be induced by heat stress [26] and high light [12]. Examination of the expression of both *APX2* and *HsfA2* provides important information into the status of the plant stress response. The most striking aspect of the gene expression data provided here is the difference between the expression pattern of *APX2* in *A*. *thaliana* and in the other species. *APX2* is very highly expressed in response to heat and high light in *A*. *thaliana*, this is consistent with other studies [23]. The five *Boechera* species have induced expression of *APX2* but the fold differences are considerably lower. These differences could be due to differences in translation efficiency and future studies should be conducted to determine if there are differences in the APX2 protein levels between *A*. *thaliana* and the *Boechera*. The expression levels of the gene Violaxanthin de-epoxidase were also examined and it was found that it did not vary across species at all, that is the *A*. *thaliana* expression levels were the same as those for the *Boechera* see S1 Fig. The gene expression studies were conducted on the same samples. This indicates that the differences in gene expression seen between *A*. *thaliana* and *Boechera* for *APX2* are real and not an experimental artifact. We can then conclude that the level of *APX2* expression is not correlated with the tolerance of a species to combined heat and high light stress. Both *B*. *californica* and *B*. *depauperata* have high tolerance to basal 43°C/HL stress (Fig 4D) and have much lower *APX2* expression (Fig 8B) than the other species studied. All of the *Boechera* species are more tolerant to a combined heat and HL pretreatment and a 43°C/HL stress (Fig 6B) than is *A*. *thaliana* despite the fact that *A*. *thaliana* has significantly higher *APX2* expression (Fig 8A). These findings also raise the question of the level of ROS in *Boechera* species during stress. Do the *Boechera* produce less *APX2* because less ROS is produced? This is an important question and future studies will examine both the levels of ROS and APX2 protein within *Boechera* cells during stress.

*HsfA2* activates many genes that are needed for cellular response to stress, including high light stress [26,27]. In this study the two species with the highest *HsfA2* expression are *A*. *thaliana* with the highest expression levels and *B*. *johnstonii* with the second highest HsfA2 expression level. We have noted above that *B*. *johnstonii* is the least tolerant of all the *Boechera* species and that *A*. *thaliana* has at best modest levels of tolerance compared to the *Boechera*. Clearly expression of the *Hsfs* is needed for stress tolerance but in this case the level of *HsfA2* expression appears to be inversely correlated with stress tolerance. This suggests that *HsfA2* expression in the species that are not highly stress tolerant may be an indication of the induction of repair mechanisms (making chaperone activity of HSPs necessary) once stress has caused damage at the cellular level. In this case, more tolerant species are not perceiving stress as highly, and therefore are not in need of the repair functions of HSPs that *HsfA2* induces.

### Species-specific stress response patterns do not strictly reflect habitat and have important implications in a warming climate

It has been suggested that comparisons of congeneric species that differ in their geographic distribution, both in latitude and elevation, are key to understanding adaptive variation and adaptive potential as global climates change [55]. For a number of reasons the *Boechera* species are an excellent model with which to study the evolution of stress responses in plants. *Boechera* is a large genus of over 70 species found in western North America [5]. The *Boechera* have recently been the subject of numerous taxonomic [1–3] and evolutionary studies [56–58], and have recently become a model system for evolutionary and ecological studies [59]. The *Boechera* contain both widespread and narrowly endemic species, and include both polyploids and diploids. The species studied here are all native to California and are found in quite different environments, see Table 1 [60]. Our study species are also a mix of polyploids and diploids. *B*. *arcuata*, *B*. *johnstonii*, and *B*. *perennans* are diploids. *B*. *californica* and *B*. *depauperata* are polyploids. The taxonomy and phylogenetic relationships among the *Boechera* are in considerable flux [1–3],[5]. At this time the phylogenetic relationships among the five species examined here is not clear, however; it is well established that they are all members of the *Boechera* genus.

The patterns of stress response across the five *Boechera* species studied are complex. These patterns become even more complex when examined in relation to species habitat and ploidy level. The most tolerant *Boechera* species is *B*. *depauperata*, a polyploid high elevation species. It is well known that polyploids often have fitness advantages over diploid relatives [61]. In addition, the ambient light intensity at an elevation of over 2500 meters is significantly higher than that at sea level. This combination of high elevation and polyploidy may partially explain the high stress tolerance demonstrated for this species. However, polyploidy and habitat do not explain all of the complex patterns seen in *Boechera* tolerance to combined stress. It is interesting that the desert species *B*. *perennans* was not among the more tolerant species. *B*. *perennans* is a perennial, as are all the other *Boechera* species, and as such it must over-summer, that is it must be able to tolerate long hot and dry summers. But this species is less tolerant to combined heat and light stress than are the chaparral species *B*. *californica*, a polyploid, and the widespread *B*. *arcuata*, a diploid. Comparisons of the tolerance of *B*. *californica* and *B*. *arcuata* reveal even more complex patterns of stress tolerance. For example, *B*. *californica* YII is more tolerant to basal stress (Fig 4) but *B*. *arcuata* YII is more tolerant to acquired stress (Figs 5–6).

Our findings have significant implications for conservation efforts focused on *Boechera* and on other rare and endangered species. Our finding that *B*. *johnstonii* has lower tolerance to combined stress than do the other *Boechera* species is important because this species has been designated as rare and endangered. *B*. *johnstonii* is found in the Peninsular Mountains of southern California at elevations of 1300-1700m. It is known from just a few populations and as such is currently at risk of extinction. As our climate warms the frequency and duration of heat waves will increase and this can put even widely distributed species at risk. The low tolerance *B*. *johnstonii* exhibits to combined heat and high light stress indicates that this species should be a high priority for conservation efforts. It also raises interesting questions about the role that stress tolerance has biogeographical patterns. Research conducted on animal tolerance to abiotic stress suggests that species that are currently near their lethal thermal limits may have a reduced capacity to respond to further environmental changes [55,62]. Our findings presented here suggest that both *B*. *perennans* and *B*. *johnstonii* are at or near their thermal limits, indicating that they are both at risk. Clearly, as global climate change continues and heat stress becomes more frequent studies of stress tolerance should be included in evaluations of species distributions and be carefully considered in conservation management priorities.

## Conclusions and Future Directions

Plant responses to stress on a molecular level are complex. As sessile organisms, plants must use numerous mechanisms and pathways in order to respond to and be tolerant of the stresses that they are subjected to in nature. In order to fully understand the relationship between multiple stress response pathways and gain a better understanding of the interactions between these pathways, utilization of various approaches to quantifying these responses is vital. This study used three different methods to assess plant response and tolerance to combined heat and high light stress: leaf damage, chlorophyll fluorescence, and gene expression. We documented extensive variation in both tolerance to stress, the ability to repair PSII after stress, and gene expression patterns in response to stress. The patterns of stress tolerance documented here among was not strictly correlated to either habitat or ploidy level. Importantly we found that the rare and endangered *B*. *johnstonii* has the lowest tolerance to combined stress. Our work illustrates the importance of conducting stress studies with groups of related species from natural populations and sets the stage for future studies that seek to uncover the mechanisms underlying the variation in stress tolerance identified among the *Boechera*.

## Supporting Information

S1 Fig Stress induced expression of violaxanthin de-epoxidase (VDE). Gene expression change calculated using the ΔΔCq method for VDE compared to reference genes and to control expression. The color legend indicates the species examined.
(EPS)
